# The effect of psychosocial support on caregivers’ perceived criticism and emotional over-involvement of persons with dementia: an assessor-blinded randomized controlled trial

**DOI:** 10.1186/s12913-019-4551-x

**Published:** 2019-10-24

**Authors:** Heidi Bjørge, Kari Kvaal, Ingun Ulstein

**Affiliations:** 1 Faculty of Health Sciences, Department of Nursing and Health Promotion, OsloMet - Oslo Metropolitan University, Pb. 4, St. Olavs plass, N-0130 Oslo, Norway; 2 Inland Norway University of Applied Sciences, Faculty of Health and Social Sciences, Elverum, Norway; 3 The Memory Clinic, Department of Geriatric Medicine, Oslo University Hospital Trust, Ullevål, Oslo, Norway; 4 Institute of Clinical Medicine, University of Oslo, Oslo, Norway

**Keywords:** Dementia, Caregiver, Psychosocial intervention, Clinical trial, Relationship quality, Expressed emotion

## Abstract

**Background:**

Many relatives of close family members suffering from dementia have taken on the caregiver role. While intervention studies have revealed promising results on caregiver burden, distress, and depression, there is a lack of knowledge about how caregivers’ perceived relationship with their ill family member influences the burden of care.

This study examined whether a psychosocial intervention influenced this perceived relationship from the caregivers’ perspective. We also explored whether the caregivers’ perception of the care receiver’s attitude and behavior changed over time, and whether caregiver stress and mood differed following the intervention.

**Methods:**

The participating caregivers and care receivers were randomly assigned to a psychosocial intervention comprising education about dementia, counselling and group sessions, or to treatment as usual. The study investigated caregivers’ experience of expressed emotion using the Felt Expressed Emotion Rating Scale (FEERS), a self-report questionnaire that captures caregivers’ perception of criticism (CC) and emotional over-involvement (EOI) exhibited by the family members with dementia.

**Results:**

A total of 208 dyads were enrolled in the study. There were no significant differences between the intervention and control groups in the studied variables. Caregivers’ perception of CC and EOI was low but fluctuated somewhat, whereas their mood and stress level were stable during the follow-up period.

**Conclusions:**

According to the FEERS, the intervention did not influence caregivers’ perception of CC and EOI, and there was no difference between the intervention and control groups regarding caregivers’ perceived relationship. Despite the increased symptoms of dementia, caregivers’ level of distress and mood remained stable, and they seemed to maintain a positive perception of the quality of their relationship with the care receiver.

**Trial registration:**

Clinical.Trials.gov Sept. 2009, NCT 01287767.

## Background

Many relatives take on the role of caregiver to support family members with dementia and enable them to live at home for as long as possible. However, caregiving has a significant impact on the caregivers’ situation, with a high risk of somatic and psychiatric health problems [1–5].

Due to increasing life expectancy and the growth of the ageing population, a growing number of family members are faced with the transition to a caregiving relationship [6]. As the progression of the disease have a duration of 3–20 years [7], the caregiving role can be a long drawn-out process. While many studies focus on the burden of care, few have investigated the impact of strain and burden on the caregiver. The neuropsychiatric symptoms associated with dementia are the main cause of caregiver distress [8–10], whereas the relationship between the caregiver and care receiver has been shown to influence the well-being of both parties. Studies have demonstrated that a poor-quality relationship may lead to further loss of functional ability and cognitive deterioration in the care receivers, while increasing strain, reducing perceived self-efficacy, and leading to depression in caregivers [11], in addition to social isolation [8, 12].

A common way of studying relationship quality is by the concept of expressed emotion (EE). EE is a psychological term that refers to the emotional climate of a person’s family environment and is derived from studies of schizophrenia. EE encompasses attitudes and behaviors that reflect caregivers’ perception of criticism and hostility from, as well as emotional over-involvement of the ill family member and has shown a strong association with the outcomes of many disorders, including dementia [13]. A high level of EE is associated with caregiver burden, distress, and depression [14–20]. In a review, Wearden et al. [13] stated that caregivers’ criticism is related to the care receivers’ repetitive speech and behavior, messiness, aggressiveness, and argumentativeness. They argue that critical caregivers may perceive this behavior as intentional and thus, controllable. However, they concluded that there is scant evidence of a predictive association between caregiver EE and the course of the illness.

Psychoeducational family interventions in studies on schizophrenia have shown beneficial effects on the relapse reduction rate, reduced family burden, and improved relationships within the family [21]. Inspired by these positive results, tailored multicomponent psychosocial interventions have been designed in dementia care and have shown promising, but small, effects on burden [22, 23], depression [24, 25], well-being and stress in caregivers [26]. Multicomponent interventions that include the person with dementia seem to be the most beneficial [25, 27–31] and may reduce the risk of early institutionalization [27].

However, none of these studies investigated whether a psychosocial intervention can change the level of perceived EE and promote a mutual relationship. An important question is whether reduced stress, depression, and time spent together influence the caregiver’s way of perceiving the care receiver’s attitude and behavior.

Only a few studies have investigated how the emotional relationship develops as dementia progresses, with contradictory results. Some authors have argued that the relationship declines, probably because of communication difficulties [32, 33]. In contrast, others have found increased closeness and mutual affection [11], perhaps because caregiving necessitates more frequent interaction [34].

Usually, the studies capture how EE influences the ill family member, whereas less attention has been given to the caregivers’ perception of their ill family members’ attitude, which is the target of this study. The present study is part of an intervention study exploring the effect of a multicomponent psychosocial intervention program on family caregivers and people with dementia. Whereas earlier published studies on the material by Bruvik and co-workers [35] found no differences in depressive symptoms between the intervention and control group, the aim of the present study was to examine whether a psychosocial intervention could influence the perceived relationship from the caregivers’ point of view. Furthermore, we assessed caregivers’ experiences of criticism (CC) and emotional over-involvement (EOI) exhibited by their family member with dementia, and whether they perceived that the attitude and behavior of the care receiver changed over time.

## Methods

### Participants

**Fig. 1:**
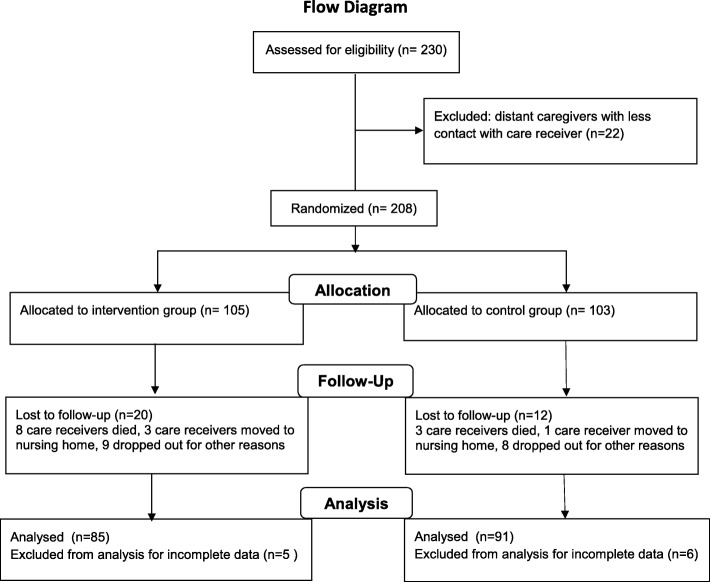
Flow diagram of the study design

We carried out an assessor-blinded multicenter randomized controlled study of persons with mild to moderate dementia and their primary caregivers. A total of 230 family caregivers of 230 persons with dementia were recruited from 19 municipalities in Norway between October 2009 and May 2011. The participating dyads were enrolled from memory clinics and through general practitioners and the local dementia team, home care offices, and adult day-care centers, and at a local educational program for caregivers. Family caregivers and their care receivers were eligible if the care receiver was living at home, fulfilled the ICD 10 criteria for dementia, and had at least weekly face-to-face contact with their caregiver. The care receivers had to have a Mini Mental State Examination (MMSE) score > 14 [36]. We excluded relatives other than spouses and children to achieve a more homogeneous material, leaving 208 in the study at baseline (Fig. 1). Due to the variety of recruitment methods, we were unable to tabulate the number of potential participants who were contacted and did not agree to participate or failed to meet the inclusion criteria.

### Randomization

The dyads were randomly assigned to participate in a psychosocial intervention program or treatment as usual. A blinded block randomization procedure with blocks of 6 dyads was carried out at each participating center. The minimum number of included dyads in the municipalities was 8. The study was carried out in small municipalities with research assistants also working as clinicians. The group leaders did not perform any follow-up of the study participants. None of the investigators knew which group the participants had been allocated to and had no access to the data.

The duration of screening prior to randomization varied from 2 to 12 months. The variation of nearly 1 year was due to the block randomization and small communities with few participants available for inclusion.

### Intervention and control condition

The intervention was conducted over a 12-month period and consisted of three components: *education about dementia, family counseling, and group meetings*. The caregivers took part in either a community-based educational program or two half-day seminars focusing on the symptoms of dementia. Counseling included five individual one-hour sessions with each family. The counseling sessions were offered during the first 3 months after inclusion and used to identify both the needs and resources within the families. A problem-solving method was employed to find new ways of coping with unmet needs and challenging behaviors. The care receiver participated in two sessions, one of which included the entire family network. The caregivers then attended six two-hour group meetings conducted twice a month. The main approach in the group meetings for the caregivers was structured problem solving, and each meeting had one specific theme. An overall goal of the intervention was to enable the caregivers to understand that the cognitive as well as the neuropsychiatric symptoms were not intentional but due to dementia, thus reducing the risk of criticism. The caregivers also learned how to set limits to avoid distress and burden.

A manual was designed based on recommended interventions [22, 30, 31, 37] and used in order to standardize both the counseling sessions and the group meetings [38]. Local nurses and occupational therapists trained in the structured approach performed the intervention. Throughout the trial they received supervision by the main investigator through telephone conferences, and took part in regular meetings, seminars, and workshops. Due to the large number of nurses and occupational therapies performing the intervention, we were unable to tabulate the number of caregivers and care receivers attending all sessions targeted to them. The dyads in the control group were informed about available services and were free to seek treatment and support in addition to any on-going care.

### Data collection and measures

Data were collected by nurses and occupational therapists who had specialized in mental health, geriatrics or dementia, and who were trained in advance to carry out structured interviews with the caregivers and care receivers. They were all blinded to the randomization.

Efficacy assessments were made at baseline and 12 months. The primary efficacy variables at the caregiver level were the Felt Expressed Emotion Rating Scale (FEERS) [39]. The FEERS is a self-rated instrument derived from the theory of EE and comprises two dimensions, namely CC and EOI. The FEERS includes three items in each cluster using a 6-point Likert scale from 0 to 5, with a higher total score indicating the perception of a more critical and emotionally over-involved attitude from the care receiver. The scale was originally developed to capture the care receivers’ perception of the caregivers’ level of EE. However, in this study we used the scale to explore how the caregiver experienced the care receiver. The FEERS was previously used in a study of dementia with acceptable internal validity (Cronbach α: CC 0.52, EOI 0.82, and factor analysis CC: 0.64–0.75, EOI: 0.73–0.80).

The Relatives’ Stress Scale (RSS) [40] is a self-rated 15-item scale that measures the caregiver’s burden of care ranging from 0 to 60, with a higher score indicating a higher degree of burden. The scale has been validated with good internal validity (Cronbach α 0.90) [40, 41].

Depression was measured using the Geriatric Depression Scale (GDS) [42], a self-rated 30-item scale ranging from 0 to 30 with or without symptoms present. A higher score indicates more symptoms of depression.

The primary efficacy variables for care receivers included neuropsychiatric symptoms measured by means of the Neuropsychiatric Inventory (NPI-Q) [43], a 12-item proxy based questionnaire focusing on the presence or absence of symptoms (yes/no). A higher score indicates more symptoms, with a total possible score of 12. The Cornell [44] was used to capture depressive symptoms. The scale is a proxy-based instrument consisting of 19 items with a score of 0–2 for each item. A higher score indicates more severe symptoms, with a possible total score of 38.

The function of activities of daily living was measured using the Instrumental Activity of Daily Living (IADL) [45, 46], which assesses the skills needed for independent living, such as the ability to do housekeeping, shopping, and manage finances. The scale has eight items with a maximum score of 3 to 5 and a total severity score of 7 to 31. A higher score indicates more need for assistance.

Cognitive function was measured using the MMSE [36]. This is an investigator-assessed score ranging from 0 to 30, with a lower score indicating more severe cognitive dysfunction.

Caregiver and care receiver demographics were collected at baseline.

### Sample size

In the power calculation, we assumed no change for the control group and an increase of at least 1 point in the intervention group for the FEERS CC and EOI subscales after the intervention. With a power of 80% and significance level of 5%, we would need 64 individuals in each group (given the anticipated difference of 1 point and common SD of 2 points). We included 105 and 103 in the intervention and control groups, respectively, at baseline. Thus, our study was sufficiently powered.

### Statistical analysis

Care receiver and caregiver characteristics at baseline were compared between the intervention and control groups using a two-tailed t-test for continuous variables and two-tailed Mann-Whitney U test for skewed data; χ^2^ was used for categorical variables.

Missing item scores were replaced by the total mean when ≤20% of scores were missing on the questionnaires.

We calculated the difference in the change in FEERS CC, FEERS EOI, and FEERS total from baseline to follow-up for the intervention and control groups using χ^2^. Possible differences between the intervention and the control group regarding the outcome variables over time were modeled using linear mixed models for repeated measures. The results are expressed as the estimated regression coefficients B with 95% CI (confidence intervals). All tests were two-sided. *P* ≤ 0.05 was considered significant. Statistical analyses were performed using the statistical program SPSS version 23.

## Results

Of the 208 dyads included in this study, thirty-two were lost to follow-up: 11 because the care receivers died, 4 because the care receiver was moved to a nursing home, while the reason for the loss of the other 17 were not registered. Among the caregivers lost to follow-up, 13 were wives or cohabitees, 5 husbands and 14 were adult children, thus leaving 40 wives or cohabitees, 11 husbands, 27 daughters and 7 sons for the intervention group, and 34 wives or cohabitees, 15 husbands, 34 daughters and 8 sons for the control group. No statistically significant differences were found between caregivers and care receivers lost to follow-up (*N* = 32) compared to those who remained in the study (*N* = 176) with regard to socio-demographic data at baseline (data not shown).

The background characteristics of the 208 caregiver - care receiver dyads are reported in Table 1.

**Table 1:** Baseline characteristics of the 208 caregivers and care receivers

	Intervention (*n* = 105)	Control (*n* = 103)
Caregivers		
Females, n (%)	81 (77)	77 (75)
Males, n (%)	24 (23)	26 (25)
Spouses, n (%)	63 (60)	55 (53)
Children, n (%)	42 (40)	48 (47)
Age, mean (SD) range	64.1 (12.2) 35–87	63.6 (11.8) 40–89
Living together, n (%)	66 (63)	56 (54)
Daily contact, n (%)	69 (66)	59 (57)
RSS, mean (SD)	25.05 (10.88)	23.48 (11.07)
GDS, mean (SD)	7.70 (6.62)	5.69 (5.70)
FEERS CC, mean (SD)	3.78 (2.82)	3.42 (2.99)
FEERS EOI, mean (SD)	6.12 (3.26)	5.91 (3.19)
FEERS Total, mean (SD)	9.95 (4.94)	9.33 (4.64)
Care receivers		
Females, n (%)	55 (52)	56 (54)
Males, n (%)	50 (47)	47 (46)
Age, mean (SD) range	78 (7.5) 60–94	79 (7.0) 58–94
Years of symptoms, median (IQR) range	3 (2, 5) 0.2–14	4 (2.5, 6) 0.6–12
Years of schooling, median (IQR) range	9 (7, 12) 3–22	9 (7, 11.3) 6–22
Formal assistance, n (%)	74 (71)	73 (50)
MMSE, mean (SD)	20.9 (3.52)	21.5 (3.68)
NPI, mean (SD)	4.8 (2.43)	5.4 (3.68)
IADL, mean (SD)	22.2 (7.72)	21.4 (7.72)
Cornell, mean (SD)	8.02 (5.54)	7.3 (5.90)

*IQR* Interquartile range, *FEERS CC* Felt Expressed Emotion Rating Scale; criticism, range 0–5. *FEERS EOI* Felt Expressed Emotion Rating Scale; emotional over involvement, range 0–5. *RSS* Relatives’ Stress Scale, range 0–12, *GDS* Geriatric Depression Scale, range 0–30, *MMSE* Mini Mental State Examination, range 0–30, *NPI* Neuropsychiatric Inventory (only presence of symptoms, range 0–12), *IADL* Instrumental Activities of Daily Living, range 7–13. Cornell, range 0–38

The characteristics of the dyads in the intervention group were comparable to those of the control group except for caregiver depression with a significantly higher GDS-score in the intervention group (*p* = 0.02), and neuropsychiatric symptoms (NPI) were lower (*p* = 0.08) in the intervention group than the control group. The FEERS items did not differ significantly between the intervention and control groups from baseline to follow-up and the FEERS CC, FEERS EOI, and FEERS total were quite stable over the 12-month period (Table 2).

**Table 2:** Linear mixed model for repeated measures of 208 caregiver/care receiver dyads assessed at baseline and 12-month follow-up (intervention *N* = 105, control *N* = 103)

Variable	Baseline score	Follow-up score	Between-group differences at follow-up (95% CI)	*P*-value
Caregivers				
RSS				
Intervention	25.0	24.9	1.12 (−1.23 to 3.48)	0.35
Control	23.5	23.9		
GDS				
Intervention ^a^	7.7	7.6	1.70 (0.41 to 3.0)	0.01
Control ^a^	5.7	6.2		
FEERS CC				
Intervention	3.8	3.3	0.16 (− 0.41 to 0.73)	0.59
Control	3.4	3.4		
FEERS EOI				
Intervention	6.1	6.5	0.17 (−0.5 to 0.84)	0.61
Control	5.9	6.3		
FEERS TOTAL				
Intervention	10.0	9.8	0.36 (−0.62 to 1.34)	0.47
Control	9.3	9.7		
Care receivers				
MMSE				
Intervention ^a^	21.0	17.5	−0.78 (−1.78 to 0.22)	0.12
Control ^a^	21.5	18.5		
NPI				
Intervention ^a^	4.8	5.0	−0.29 (−0.83 to 0.26)	0.31
Control ^a^	5.4	4.9		
IADL				
Intervention	22.2	25.0	0.48 (−0.73 to 1.69)	0.44
Control	21.4	24.6		
Cornell				
Intervention ^a^	8.2	7.5	0.34 (−0.81 to 1.49)	0.57
Control ^a^	7.9	7.1		

Positive values reflect a decrease in scores compared to baseline. ^a^ = Some missing data. *FEERS CC* Felt Expressed Emotion Rating Scale; criticism, range 0–5. *FEERS EOI* Felt Expressed Emotion Rating Scale; emotional over involvement, range 0–5, *RSS* Relatives’ Stress Scale, range 0–6, *GDS* Geriatric Depression Scale, range 0 to 30, *MMSE* Mini Mental State Examination, range 0–30, *NPI* Neuropsychiatric Inventory (only presence of symptoms, range 0–12), *IADL* Instrumental Activities of Daily Living, range 7–13. Cornell, range 0–38

The living conditions of the care receivers changed during the follow-up period, as 24% moved to nursing homes, 24 from the intervention group and 23 from the control group. Nursing home placement was not associated with any of the FEERS items.

We assessed whether the time from screening to randomization had any influence on the caregivers’ perceived relationship and found no significant differences between caregivers who had to wait (*n* = 14) and those who did not have to wait (*n* = 162) (data not shown).

## Discussion

Although the intervention was based on multicomponent strategies that have shown the best results on health and well-being, quality of life, and mood [30, 31], there were no significant differences between the intervention and control groups regarding the caregivers’ perceived relationship.

In accordance with our findings, Wearden et al. [13] found that the level of EE in dementia is low compared to other illnesses. Thus, a further decrease in EE may be unlikely. In contrast to most other studies in which the level of EE is ascertained from the caregivers’ attitude, our study captured caregivers’ perception of the care receivers’ attitude and behavior from the caregivers’ own perspective. To the best of our knowledge, only one earlier study in dementia addressed caregivers’ perception of criticism from their care receiver. In that study, caregivers described their relationship as warm and relatively free of conflicts and criticism [47], a finding that is consistent with our study.

Even though we excluded more distant relatives, the participants were still fairly heterogeneous with respect to kinship, distress, and emotional relationship to the care receiver, which could also have influenced the results. Older spouses may be frailer and more vulnerable to stress [48], whereas adult children often find themselves balancing care obligations for their ill parent and demands from their own family and work. For elderly wives, caring for a husband may also be a greater physical challenge.

The caregivers in this study seemed to perceive a good mutual relationship, as shown by the low level of FEERS CC and FEERS EOI. Their experience of the care receiver having a positive attitude towards them did not change significantly over time. Nor did the intervention influence the caregivers’ perception of the relationship. What changed during this period was the care receivers’ functional and cognitive capacity, which declined. Although this decline would be expected to influence the caregivers’ health and well-being [2, 3, 5], their level of stress and emotional status did not change over an extended period of time. In accordance with the review by Wearden et al. [13], a decline in cognitive or ADL-function did not influence caregivers’ EE. This may indicate that, when caregivers perceive a positive relationship with their ill family member, the caregivers are not affected by the decline in their ill family member’s health [32].

The gradual loss of a functioning spouse or parent involves emotional and physical challenges for both spouses and adult children. As the care receivers’ cognitive and functional abilities decline, this gradual loss of their former relationship can be seen as an ambiguous loss [49], the loss of the person they once knew although they are still physically present. Along with their own strain and mourning their own loss, they must adjust to the changing needs of their ill family member. To some degree, caregivers’ perceptions of the relationship quality and distress were stable, and the extent to which EE is a trait-like aspect and related to personality has been discussed previously [13, 50]. Due to the progressive nature of dementia, we could have expected higher levels of stress, making caregivers perceive the care receiver as having higher EE. However, the caregivers in our study still perceived their family members’ attitude as positive at the 12-month follow-up, which might be seen in accordance with the study by Hooley [50] where it is argued that characteristics of greater tolerance and flexibility were related to caregivers with low EE. Although related to caregivers’ EE, this might be relevant for caregivers’ tolerance in our study. The Danish philosopher Løgstrup [51] argued that caring is a fundamental factor in humanity; thus, taking care of an ill family member seems to be a natural consequence. In addition, there may be increased knowledge and insight over time that the family member’s behavior is not intentionally controlled but related to the disease. For example, studies of schizophrenia have found that EE fluctuates with symptoms, with higher EE in acute phases. The gradual decline in cognitive and functional capacity in our study did not seem to influence the caregivers’ perception of the quality of their relationship. This finding is in line with the study by Vitaliano et al. [20], who found that the EE status in spouses was stable during a 15 to 18-month period even though they were not offered any intervention.

As recorded in the initial dataset, half of the caregivers had a score > 24 on the RSS, indicating an increased risk of depression [52] and the need for more specific approaches to prevent or alleviate the symptoms of depression. A problem-solving approach was used in the counseling and group sessions, with the intention of focusing on how to solve daily life challenges. However, a comprehensive program directed at how to reduce depressive feelings may have been more appropriate for the most distressed caregivers, which is in line with today’s recommendations for individualized caregiver interventions.

The strength of the study is the large number of participants. The power was good enough to reduce the risk of type II error, which overlooks a positive result. However, a limitation is the fact that those who declined to participate were not mapped. Thus, we do not know if those who took part in the study are representative of relatives of people with dementia in terms of burden and distress. Only the less distressed caregivers and care receivers may have agreed to participate, thus limiting the ability to generalize the findings. However, the level of distress in the sample did not differ significantly from other studies [52], which reduces the risk of limiting the generalizability. Another obvious limitation is the surprisingly low level of EE in this sample of home dwelling care receivers, and factors that, in terms of experience, lead to high EE. Retrospectively one can ask whether other objectives and measuring instruments should have been chosen. Perhaps the low level of EE reveals that the FEERS is not a sensitive enough instrument to catch changes in caregivers’ perceived EE.

Another challenge in performing studies in which the respondents are interviewed is the so-called Hawthorne effect [53]. The caregivers in the control group may experience the interview situation as an opportunity to put their burden into words and perceive the interview as a pleasant event with a therapeutic effect.

## Conclusions

Low FEERS CC and EOI scores over time indicated a better mutual relationship between caregiver and care receiver than expected. The decline in care receivers’ functional and cognitive capacity highlights a willingness to care, often at the caregiver’s own expense. As no differences were found between the intervention and control groups regarding the caregivers’ perceived relationship, further studies would be needed to find individually tailored interventions taking into consideration caregivers’ perceived relationship quality and their own level of stress. By using the FEERS, which offers a quick and easy assessment of the relationship as perceived by caregivers, clinicians would be able to pinpoint caregivers and patients in need of an approach focusing on how to improve a perceived relationship characterized by a high level of EE.

## Data Availability

The dataset used and analyzed during the current study are available from the corresponding author on request.

## Abbreviations

CC: Critical comments
EE: Expressed Emotion
EOI: Emotional over-involvement
FEERS: Felt Expressed Emotion Rating Scale
GDS: Geriatric Depression Scale
IADL: Instrumental Activities of Daily Living
MMSE: Mini Mental State Examination
NPI-Q: Neuropsychiatric Inventory
RSS: Relatives’ Stress Scale
χ^2^: Chi-Square test statistic
